# Quantification of human plasma metalloproteins in multiple sclerosis, ischemic stroke and healthy controls reveals an association of haptoglobin-hemoglobin complexes with age

**DOI:** 10.1371/journal.pone.0262160

**Published:** 2022-01-12

**Authors:** Sophia Sarpong-Kumankomah, Katherine B. Knox, Michael E. Kelly, Gary Hunter, Bogdan Popescu, Helen Nichol, Karen Kopciuk, Henry Ntanda, Jürgen Gailer

**Affiliations:** 1 Department of Chemistry, University of Calgary, Calgary, Canada; 2 Department of Physical Medicine and Rehabilitation, College of Medicine, University of Saskatchewan, Saskatoon, Canada; 3 Division of Neurosurgery, College of Medicine, University of Saskatchewan, Saskatoon, Canada; 4 Division of Neurology, College of Medicine, University of Saskatchewan, Saskatoon, Canada; 5 Department of Anatomy, Physiology and Pharmacology, University of Saskatchewan, Saskatoon, Canada; 6 Departments of Mathematics and Statistics, Oncology and Community Health Sciences, University of Calgary and Cancer Epidemiology and Prevention Research, Cancer Control Alberta, Calgary, Canada; 7 Department of Pediatrics, Cumming School of Medicine, University of Calgary, Calgary, Canada; East China Normal University School of Life Sciences, CHINA

## Abstract

Advanced analytical methods play an important role in quantifying serum disease biomarkers. The problem of separating thousands of proteins can be reduced by analyzing for a ‘sub-proteome’, such as the ‘metalloproteome’, defined as all proteins that contain bound metals. We employed size exclusion chromatography (SEC) coupled to an inductively coupled plasma atomic emission spectrometer (ICP-AES) to analyze plasma from multiple sclerosis (MS) participants (n = 21), acute ischemic stroke (AIS) participants (n = 17) and healthy controls (n = 21) for Fe, Cu and Zn-metalloproteins. Using ANOVA analysis to compare the mean peak areas among the groups revealed no statistically significant differences for ceruloplasmin (p = 0.31), α_2_macroglobulin (p = 0.51) and transferrin (p = 0.31). However, a statistically significant difference was observed for the haptoglobin-hemoglobin (Hp-Hb) complex (p = 0.04), being driven by the difference between the control group and AIS (p = 0.012), but not with the MS group (p = 0.13), based on Dunnes test. A linear regression model for Hp-Hb complex with the groups now adjusted for age found no statistically significant differences between the groups (p = 0.95), but was suggestive for age (p = 0.057). To measure the strength of association between the Hp-Hb complex and age without possible modifications due to disease, we calculated the Spearman rank correlation in the healthy controls. The latter revealed a positive association (r = 0.39, 95% Confidence Interval = (-0.05, 0.83), which suggests that either the removal of Hp-Hb complexes from the blood circulation slows with age or that the release of Hb from red blood cells increases with age. We also observed that the Fe-peak corresponding to the Hp-Hb complex eluted ~100 s later in ~14% of all study samples, which was not correlated with age or disease diagnosis, but is consistent with the presence of the smaller Hp (1–1) isoform in 15% of the population.

## 1. Introduction

Disease specific molecular biomarkers can be quantified in a variety of human bodily fluids, including urine, saliva, cerebrospinal fluid and blood [1]. Among these biofluids, plasma is one of the most information-rich [2] and its analysis is particularly relevant from an analytical point of view as it receives biomolecules that are released from specific organs and/or red blood cells as a result of disease processes [3–5]. Since plasma is easily accessible and may harbor diagnostically relevant biomarkers [6–9], the development of advanced methods for specific molecular biomarkers is a vibrant area of research [10]. Plasma, however, also represents one of the most challenging proteomes to characterize analytically as it contains thousands of proteins [11], both abundant and rare, which span a dynamic concentration range of 10 orders of magnitude [2]. One protein in plasma that plays an important role in health and disease is the α_2_-sialoglycoprotein haptoglobin (Hp). Hp is an acute phase protein which strongly binds to hemoglobin (Hb) that is released from the turnover of red blood cells and thus prevents iron loss, kidney damage and heme induced inflammation [12–14]. Total Hp is known to increase with age in rats and humans [15], but it is not known if Hp bound to Hb (Hp-Hb) increases with age. Although Hp is found in the plasma of all mammals, only humans display a Hp polymorphism which is associated with the presence of Hp isoforms 1–1 (a dimer) or the larger Hp 1–2 and Hp 2–2 isoforms [12].

The inherent difficulty of separating thousands of proteins in plasma can be significantly reduced by analyzing this analytically complex biofluid for a ‘sub-proteome’ using an appropriate analytical method. The ‘metalloproteome’ constitutes such a ‘sub-proteome’ in which Cu, Fe and Zn are bound to individual plasma proteins [16]. Analytical methods, such as size exclusion chromatography (SEC) coupled to an inductively coupled plasma atomic emission spectrometer (ICP-AES) can be used to analyze plasma for metalloproteins [17]. This technique is capable of analyzing plasma (0.5 mL) for ~12 metalloentities [16, 18, 19] and is complementary to the determination of plasma protein biomarkers with more conventional analytical methods, such as mass spectrometry-based proteomics and/or radioimmunoassay-based methods [6, 20]. Indeed, the quantification of metalloproteins, such as ceruloplasmin (Cp) in plasma can be employed to diagnose diseases, such as Wilson’s disease (WD) as Cp is essentially absent in plasma of WD patients [21]. It has also been reported that the metal concentrations of Mg, Mn, Cu and Se in human plasma from acute ischemic stroke (AIS, n = 10) were statistically different from hemorrhagic stroke (n = 9) participants [22]. Since blood is in constant contact with the brain, it is possible that the aforementioned differences may be attributed to the release of metal species to blood plasma.

In the last two decades it has indeed been established that certain neurodegenerative diseases are associated with the dyshomeostasis of essential metals, such as Fe, Cu and Zn [23], which are present in gray matter at concentrations between 0.1–0.5 mM [24, 25]. Several studies have demonstrated that these metal ions are directly implicated in the pathophysiology and pathogenesis of Alzheimer’s disease [26–28], multiple sclerosis (MS) [29–31] and stroke [32]. To evaluate the potential that the quantification of plasma metalloproteins may offer in terms of disease diagnosis, progression, or severity, we conducted a pilot study in which we quantified Fe, Cu and Zn-containing metalloproteins in plasma collected from AIS and MS participants (disease control) as well as healthy controls. Although the statistical analysis of individual metal-containing metalloprotein peak areas did not reveal significant differences between the plasma from the respective groups, we observed a statistically significant correlation between age and an Fe-peak corresponding to a Hp-Hb complex. In addition, a markedly different retention time for a Hp-Hb complex was observed in about 15% of participants in each group. In our previous work this Fe-peak was identified as a Hp 1–1 isoform in these individuals [12].

## 2. Materials and methods

### 2.1. Chemicals and solutions

Phosphate buffered saline (PBS, 150 mM, 1 pouch powder prepares 1 L) and an indium standard (1000 mg/L ± 0.003 in 2% HNO_3_) were purchased from Sigma-Aldrich (St. Louis, MO, USA), while HCl (plasma pure 34–37%), plasma-pure plus HNO_3_ (67–70%) as well as Fe, Cu and Zn standards (plasmaCAL, 1000 ppm) were purchased from SCP Science (Baie D’Urfe, Quebec, Canada). PBS buffer mobile phase was prepared using DI water from a Simplicity water purification system (18.3 MΩ●cm, Millipore, Billerica, MA, USA) followed by the adjustment of the pH using a VWR Symphony SB20 pH meter (Thermo Electron Corporation, Beverly, MA, USA) and filtration through 0.45-μm nylon-filter membranes (Mandel Scientific, Guelph, ON, Canada). The SEC column was size calibrated using a protein mixture (Bio-Rad Laboratories, Hercules, CA, USA) which contained thyroglobulin (670 kDa), ɣ-globulin (158 kDa), ovalbumin (44 kDa), myoglobin (17 kDa) and vitamin B12 (1.35 kDa). The acid digestion procedure for the analysis of select serum samples for total Cu, Fe and Zn was validated using the standard reference material DOLT-5 (dried dogfish powder, certified for Fe– 1070 mg/kg ± 80; Zn 105.3 mg/kg ± 5.4 and Cu 35.0 mg/kg ± 2.4) which was obtained from the National Research Council of Canada (Ottawa, ON, Canada).

### 2.2. Collection of plasma samples

This study conformed with the Tri- Council Policy Statement: Ethical Conduct for Research Involving Humans (TCPS2). Ethics approval was obtained by the University of Saskatchewan (Protocol # MB-MSAS-01) (PI, H. Nichol) and written consent was obtained from all participants (age range 18–70 years) before blood was collected. Exclusion criteria for participation in the study included a history of diabetes, malignancy, angina, or any disorders of the blood. Blood plasma was obtained from 21 relapsing remitting MS participants (14 females, 7 males) within four months of an acute MS relapse confirmed by a neurologist, 17 AIS participants (5 females, 12 males) who had not received thrombolytic drugs and whose blood plasma was collected on admission to the hospital and 21 healthy controls (13 females, 8 males). While control (mean age ± standard deviation) was 41.2 ± 11.2 and MS participants was 40.9 ± 10.3 were successfully age and sex matched, however, due to the mean age of the stroke participants of 55.2 ± 11.0 we were not able to successfully match for age for AIS participants. Blood collection was performed at Saskatoon City Hospital and Royal University Hospital (Saskatoon SK) using the same type of blood collection tubes (light green BD VacutainerTM Plastic Blood Collection Tubes–PST Plasma Separation Tubes Hemogard^TM^; polymer gel/Lithium heparin additive) and the same procedure. The MS group was an appropriate disease control for brain involvement since acute MS lesions typically are inflammatory and are similar to ischemic stroke lesions in that both display an altered iron metabolism.

A clinical iron panel (Fe, total iron-binding capacity, Tf saturation and ferritin) was completed for each patient plasma sample by the Royal University Hospital clinical lab, but the data were not relevant to our current analysis of haptoglobin-hemoglobin. Plasma samples (0.4–3.0 mL per sample) were frozen as soon as possible and stored at -80°C until sample collection was completed. Then all samples were shipped to Calgary on dry ice for analysis. Of the recruited participants, 6 samples were removed for various reasons (plasma sample lost, or clinical iron panel not done). One plasma sample was turbid after thawing and since centrifugation did not clear the solution it was not analyzed as it would have otherwise clogged/damaged the SEC column. SEC-ICP-AES was employed to analyze all plasma samples for the Cu metalloprotein ceruloplasmin (Cp), the Zn metalloprotein α_2_macroglobulin (α_2_M) and two Fe metalloproteins, namely holo-transferrin (Tf) and a Hp-Hb complex. While all plasma samples were analyzed by SEC-ICP-AES, those samples with >200 μL serum left were also analyzed for total Fe by ICP-MS in order to establish the Fe recovery after SEC-ICP-AES analysis (see below).

### 2.3. Instrumentation

#### 2.3.1. SEC-ICP-AES

The analytical SEC-ICP-AES system has been described previously [19]. Briefly, a Superdex^TM^ 200 Increase 10/300 GL (30 x 1.0 cm I.D., 8 μm particle size) high resolution SEC column was used in conjunction with a PBS buffer mobile phase (pH 7.4) and a flow rate of 0.75 mL/min (column temperature: 22°C). To estimate the molecular weight of the detected metalloprotein peaks the SEC column was calibrated by injecting a size calibration standard protein mixture (carbon emission line at 193.091 nm). All elements of interest were simultaneously detected in the column effluent by means of a Prodigy high-dispersion, radial-view ICP-AES (Teledyne Leeman Labs, Hudson, NH, USA): Cu at 324.754 nm, Fe at 259.940 nm and Zn at 213.856 nm. A 360 s delay was implemented between the injection of each plasma sample and a data acquisition rate of 1 data point per 2 s was used.

#### 2.3.2. ICP-MS

A PlasmaQuant (PQ) Elite Inductively Coupled Plasma Mass Spectrometer from Analytik Jena (Jena, Germany) was used for the analysis of the digested serum samples for total Fe, Cu and Zn. The Ar plasma flow as 9.00 L/min, the auxiliary Ar gas flow was 1.20 L/min, the sheath Ar gas flow 0.00 L/min, the nebulizer flow 0.98 L/min and the sampling depth 5.00 mm. The utilized power was 1.25 W, the pump rate was 14 rpm and the stabilization delay was 15 s.

### 2.4. Sample preparation

Plasma samples were thawed at room temperature for 45 min and each sample (750 μL) was diluted 3:1 with PBS buffer and after gentle mixing 500 μL were injected onto the SEC-ICP-AES system. In case the plasma volume for an individual was <750 μL, an equivalent volume of PBS-buffer was added to plasma to achieve a 3:1 dilution and 500 μL were injected.

### 2.5. Chromatographic data analysis

The chromatographic raw data (Salsa software version 3.0) for the time-resolved emission lines of Cu, Fe and Zn were imported into SigmaPlot 13.0, smoothed using the bi-square algorithm and the retention times and peak areas (in area units or AU) were determined using Origin Pro 2017. With regard to Cu, the area of a single Cu peak which corresponded to Cp is reported, while for Fe the peak areas corresponding to the Hp-Hb complex and Tf are reported. With regard to Zn only the peak area for the first eluting Zn peak, which corresponds to α_2_M is reported.

### 2.6. Evaluation of SEC column integrity

The consecutive analysis of ~60 human plasma samples by SEC-ICP-AES required us to ascertain that the SEC column separation efficiency was not compromised. We therefore injected rabbit plasma aliquots from a homogenous stock before and after the analysis of human plasma samples on each day. We found that the retention times of the major Fe, Zn and Cu peaks did not change over the course of the study and these findings were substantiated by the injection of a size calibration mixture (Bio-Rad Laboratories Hercules, CA, USA) twice a day. Another measure of the column integrity which was part of the SEC-ICP-AES system is to calculate the recovery of Fe and Zn after a rabbit plasma stock is analyzed. To this end, rabbit plasma was injected without a column and with a column and the emission lines of Fe and Zn were simultaneously monitored. The subtraction of the areas that were obtained for Fe and Zn with a column from the area counts that were observed without a column allowed to calculate the metal recovery, which was 37–87% for Fe (S1 Fig) and 48–107% for Zn (S2 Fig). Similar recovery results were observed for the human plasma samples as both biological fluids were analyzed at the same time (see below).

### 2.7. Statistical analysis of metalloprotein peak areas

Descriptive statistics, Spearman rank correlation coefficients, one-way analysis of variance and linear regression models adjusted for age were conducted to compare the three participant groups for individual and total Fe, Cu and Zn peak areas. Model assumptions and fits were assessed with transformations, such as taking the square root were employed to meet the normality assumptions. The influence of outliers was also evaluated by removing them and refitting the models. Analyses were carried out using the R software program (Version 4.0.6, R Core Team, 2021, R-Foundation for Statistical Computing, Vienna, Austria; https://www.R-project.org/) and Stata Statistical Software (StataCorp. 2013: Release 13, College Station, TX: StataCorp LP). A p-value of <0.05 was considered statistically significant and evidence of biological differences.

### 2.8. Validation of the microwave digestion procedure for human plasma

A standard reference material from the National Research Council of Canada (DOLT-5) was used to validate a microwave digestion procedure for the analysis of plasma. To accomplish this, 3.0 mL of conc. HNO_3_ (plasma-pure plus) were added to an aliquot of DOLT-5 (300 mg) and the mixture was digested with the Discovery SP-D microwave digestion system (CEM, Matthews, NC, United States). A digestion method was used that was a slight modification of a method that was recommended by the manufacturer. The digestion method involved a temperature of 200°C, a pressure of 400 psi, a power of 300 W, a ramp time of 7.00 min and a hold time of 9:00 min (total run time: 17 min). The digested clear and yellowish digestates (triplicate analysis) were transferred to a 10-mL volumetric flask and filled to the mark using DI water from a Simplicity UV Ultrapure Water Purification System (Millipore, Sigma). The Cu, Fe and Zn concentrations were then determined by ICP-AES using standard calibration curves.

### 2.9. Iron recovery after plasma sample analysis by SEC-ICP-AES

To determine how much of the total Fe contained in each plasma sample reached the ICP-AES detector (i.e. the Fe % recovery), we intermittently analyzed aliquots (500 μL) of a rabbit plasma stock for which the total Fe concentration was determined by ICP-MS onto the SEC-ICP-AES system without a column. This allowed us to establish how many μg of total Fe in rabbit plasma corresponded to how many Fe area counts that were obtained for each plasma sample by SEC-ICP-AES. Using these Fe peak areas and the total Fe concentration for each human plasma sample allowed us to calculate the % recovery for Fe.

### 2.10. Plasma sample analysis for total iron by ICP-MS

For those plasma samples for which excess plasma was left over, 200 μL were transferred into high purity HNO_3_-washed 10-mL acid digestion vessels and kept in a fridge until analysis. To each 200 μL plasma sample, 3.0 mL of concentrated HNO_3_ (plasma-pure plus) were added and the mixtures were digested using the validated digestion procedure. The obtained solutions were transferred to a 10-mL volumetric flask, spiked with 1 ppm In standard and filled to the mark using DI water to achieve a final concentration of 10 ppb. The latter solutions were then analyzed by ICP-MS for total Fe concentrations using standard calibration curves.

## 3. Results

### 3.1. Validation of the plasma digestion procedure

Standard calibration curves for Fe, Cu and Znobtained by ICP-AES showed an excellent linear relationship (correlation coefficients R^2^ = 0.999–1.0). The analysis of DOLT-5 digestates (triplicate analysis) by ICP-AES revealed concentrations of 1041 ± 21 mg/kg for Fe, 111 ± 2 for Zn and 35 ± 0.2 mg/kg for Cu, which corresponds to 97, 104 and 101% of the certified values, respectively.

### 3.2. Iron recovery after plasma sample analysis by SEC-ICP-AES

Standard calibration curves for Fe, Cu and Zn obtained by ICP-MS showed excellent linear relationships (R^2^ = 0.9984–0.9996). The Fe concentration range in the plasma samples was 1292–118901 ppb for ^56^Fe, 451–11477 ppb for ^66^Zn and 913–2502 ppm for ^63^Cu. The analysis of rabbit plasma stock (which was used for the column integrity check) revealed concentrations of 3535 ± 74 ppm for ^56^Fe, 1358 ± 5 for ^66^Zn and 983 ± 86 ppm ^63^Cu. The iron % recovery values (i.e. the fraction of Fe that eluted from the column compared to the total Fe injected) for the individual plasma samples (Table 1) displayed an average of 70 ± 23% for MS participants (n = 18), 68±11% for AIS participants (n = 7) and 86±21 for healthy controls (n = 12). These values are comparable to those obtained for Fe in the rabbit plasma stock.

**Table 1:** Fe recovery of serum samples after SEC-ICP-AES analysis. Using the SEC-ICP-AES area counts for Fe that were obtained for the analysis of a rabbit plasma stock for which the total Fe concentration was known allowed us to calculate how many μg of Fe injected corresponded to what total Fe area count. This correlation was then used to calculate the Fe recovery in % by using the total Fe of each serum sample (determined by ICP-MS) and the Fe area counts obtained after SEC-ICP-AES analysis.

Sample Label	SEC-ICP-AES derived Fe peak area for rabbit plasma without column (c/s)	Volume of sample serum (μL) digested	Total ^56^Fe in digested serum (ppb) by ICP-MS	Total Fe peak area (c/s) by SEC-ICP-AES	Percent recovery for ^56^Fe
2162	43479	200	2876	14692	42
2707	43479	200	1765	9785	45
2479	43479	200	1418	7764	45
2014	43479	200	2131	12725	49
3408	43479	200	2002	8749	36
1849	37702	200	2265	15849	66
1747	37702	200	2223	18863	80
2398	37702	200	3787	21625	54
3318	37702	200	3541	26199	70
2166	34559	200	1545	13652	91
1436	34559	200	2821	23857	87
3533	34559	200	1945	9963	53
3294	34559	200	1719	11095	66
2111	34559	200	1205	10689	91
1512	34559	100	2977	24799	86
2540	34559	100	3055	20616	69
2750	34559	200	2200	11835	55
3379	37731	200	2339	18756	76
3127	37731	200	2482	28497	108
3012	37731	200	3240	25588	74
1596	31197	200	2201	18756	97
2763	31197	100	4084	22703	63
2512	31197	200	2039	17999	101
2200	31197	200	2305	20151	100
2206	31197	200	1391	13201	108
2870	31197	200	2049	13669	76
3116	31197	200	1888	18483	112
2440	31197	100	1889	14795	89
1777	31197	200	4639	42568	105
2499	30371	140	3451	23476	80
2403	30371	200	2566	18733	86
2118	30371	200	1292	16788	*152
2545	30371	100	2379	11755	58
3014	30371	200	2617	22656	101
2127	30371	200	2186	17645	95
2676	30371	130	3458	21019	71
3001	30371	200	2188	28217	*151
2659	30371	200	1471	4809	38
3130	30371	200	2092	8263	46
Rabbit plasma (N = 4)	38368 ± 3220	200	3559 ±71	28371 ± 2110	± 1

*Data was not included in the calculation of the mean and standard deviation.

### 3.3. Fe-specific chromatograms of plasma samples obtained by SEC-ICP-AES

**Fig 1:**
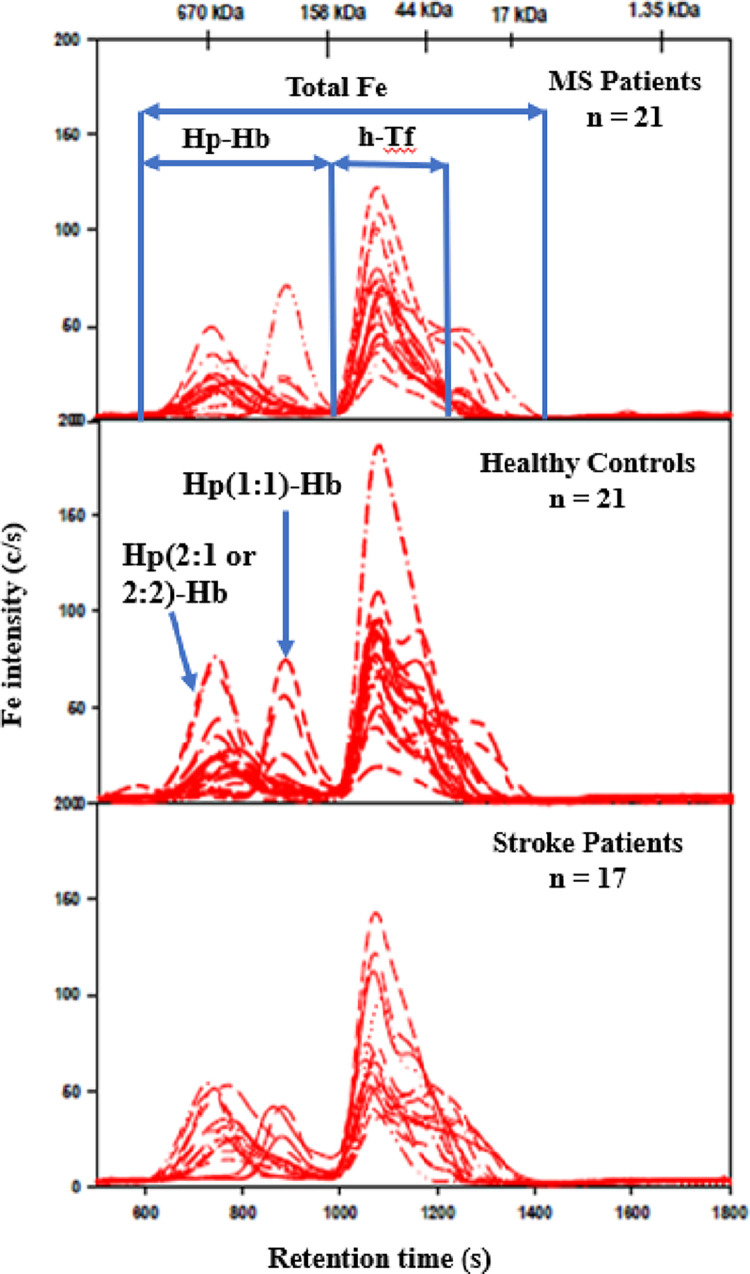
Fe-specific chromatograms obtained after the analysis of plasma from MS participants, AIS participants and healthy controls. Column: Superdex 200 Increase 10/300 GL (30 x 1.0 cm I.D., 8 μm particle size), Temperature: 22°C, Mobile phase: 150 mM PBS buffer (pH 7.4), Flow rate: 0.75 mL/min, Injection volume: 500 μL. ICP-AES detection at 259.940 nm (Fe). Retention times of molecular weight markers are depicted on top. 750 μL of plasma was diluted with 250 μL of PBS buffer and 500 μL of the obtained solution were injected on the SEC column.

In the Fe-specific chromatograms, two peaks were detected in all plasma samples (Fig 1), which is in accord with previous results using the same SEC column [16, 19]. Based on our previous studies, the Fe-peak having a retention time of 763 ± 20 s is assigned to a Hp-Hb complex and the Fe-peak with a retention time of 1,073 ± 13 s to hTf (79.7 kDa). The Fe-specific chromatograms for ~3 individuals in each group (~14%) showed that the Fe-peak corresponding to a smaller Hp-Hb complex (its retention time which was increased by ~100 s compared to the other individuals) (Fig 1). We have previously reported that the addition of different Hp isoforms (Hp 1–1, Hp 1–2, Hp 2–2) to red blood cell lysate followed by SEC-ICP-AES analysis (using the same SEC column as in the present study) resulted in an Fe-peak that corresponded to a Hp-Hb complex which had a ~100 s larger retention time for Hp 1–1 compared to Hp 1–2 and Hp 2–2 [19]. The observation that ~14% of individuals displayed a different retention time for the Hp-Hb complex in each group can therefore be explained by the genetic polymorphism of Hp [12, 19, 33]. Hp-Hb complexes are formed in plasma that differ in their size/hydrodynamic radius and thus result in different retention times. Therefore, the Hp-Hb complex which eluted with a retention time that was 100 s larger contains the Hp isoform 1–1, while all other individuals in each group predominantly contained either Hp isoform 2–1 and/or Hp 2–2. The observed frequency of this Hp isoform was similar in all groups, which is in good accord with what is expected for a Canadian population [12].

Total Fe peak areas and the individual Fe peak areas that were obtained for the analysis of plasma for all groups are shown in Table 2. While the largest mean peak area for both total Fe peaks was observed for the AIS group (15,069 ± 4,473 absorption units or AU), followed by the control group (14,338 ± 4,709 AU) and the MS group (12,100 ± 4,494 AU), no statistically significant difference was observed between these groups by one-way ANOVA, F (2, 55) = 2.25, p = 0.12. With regard to the Tf peak, the largest mean Fe-peak area was observed in the control group (10,478 ± 3,682 AU), followed by the AIS group (10,255 ± 4,146 AU) and the MS group (8,768 ± 3,694 AU). There was no statistically significant difference among the groups as determined by one-way ANOVA (F (2, 55) = 1.20, p = 0.309).

**Table 2:** Mean Fe values, standard deviations and minimum/maximum values obtained for the analysis of serum samples by SEC-ICP-AES for total Fe and peak areas for the Hp-Hb and the h-Tf peak.

Peak Area	Groups		
	Control	MS	AIS
**Total Fe**			
n*	21	21	17
Mean (SD^#^)	14,338 (4,709)	12,100 (4,494)	15,069 (4,473)
Min; Max	3,607; 21,373	5,823;19,649	8,321; 22,650
**Hp-Hb complex**			
n	19^&^	18^@^	17
Mean (SD)	3,361 (1,339)	2,584 (748)	4,581 (1,614)
Min; Max	807; 6,372	1,120; 3,603	2,288; 5,864
**h-Tf**			
n	21	21	17
Mean (SD)	10,478 (3,682)	8,768 (3,694)	10,255 (4,146)
Min; Max	2,800; 17,161	2,948; 16,362	4,281; 7,582

* number of individuals per group.

^#^ standard deviation.

^$^ two outliers removed.

^@^ three outliers removed.

The mean Fe-peak area for the Hp-Hb complex (no outliers removed) was highest for the AIS group (4,581 ± 1,614 AU), followed by the control group (3,361 ± 1,339 AU; 2 outliers removed) and the MS group (2,584 ± 748 AU; 3 outliers removed). Outliers were removed in the MS and control groups based on violating the ANOVA normality assumption as assessed by the Shapiro-Wilk test. With these five highest outliers removed, the statistical significance of the group differences via ANOVA (F (2,51) = 10.8) increased from p = 0.04 to p = 0.0001. Dunnetts test only identified statistically significant differences between the AIS group and the controls (p = 0.011), but not between the MS participants and the controls (p = 0.13). However, the age differences between the AIS and the other two groups needed to be taken into account to confirm these differences.

#### 3.2.1. Hp-Hb complex and age

A linear regression model using the square root of Hp-Hb values and adjusting for age found no statistically significant difference between the controls and either participant group (p = 0.95). The estimated regression coefficients (standard errors (SE)) for the AIS group vs controls was 1.92 (4.89) and for the MS participants vs controls was -6.14 (4.10), with corresponding p-values of 0.7 and 0.14, respectively. Age was close to statistical significance, with a p-value of 0.057 and an estimated regression coefficient of 0.32 (SE = 0.16).

**Fig 2:**
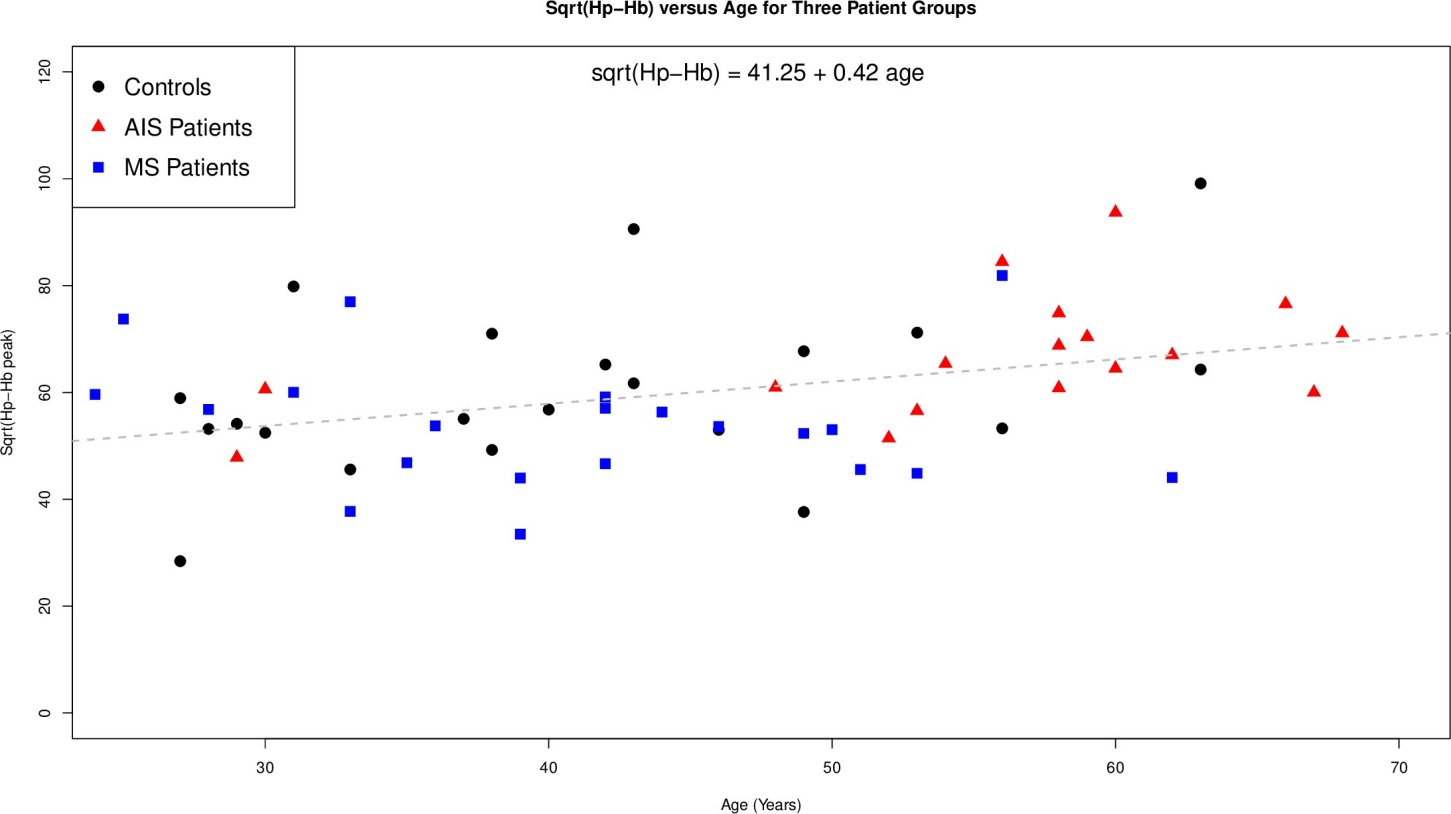
Plot of the age of the participants (AIS, MS and control: x-axis) against the square root of the obtained Fe-peak areas corresponding to the Hp-Hb complex (y-axis). The regression line is for all data (adjusted R^2^ of 0.11, F(1,57) = 8.58, p = 0.005) and ignores which group people are in, but clearly depicts a positive association between the age of the individual and the Hb-Hp peak areas.

To reveal possible trends that involve ageing, the age of participants with AIS, MS and the controls were plotted against the square root of the Hp-Hb Fe-peak area (Fig 2) to make the Hb-Hp values appear normally distributed. The Hp-Hb Fe-peak area increased in controls who had an age range that included the older AIS participants and the younger MS participants. From a linear regression model with just age regressed on the sqrt(Hp-Hb) values, explicitly ignoring the participant grouping, the sqrt(Hp-Hb) values increase by 0.42 units for every one year increase in age, which was statistically highly significant (p-value 0.005). We note that this effect is largely being driven by the AIS and the healthy control participants, but not the MS participants. To substantiate these results we also established the Spearman rank correlation value for each group, which was 0.39 [95% Confidence Interval (CI) = (-0.05, 0.83)] for healthy controls and 0.54 [95% CI = (0.03, 0.96)] for the AIS group and -0.31 [95% CI = (-0.86, 0.24)] for the MS group.

**Fig 3:**
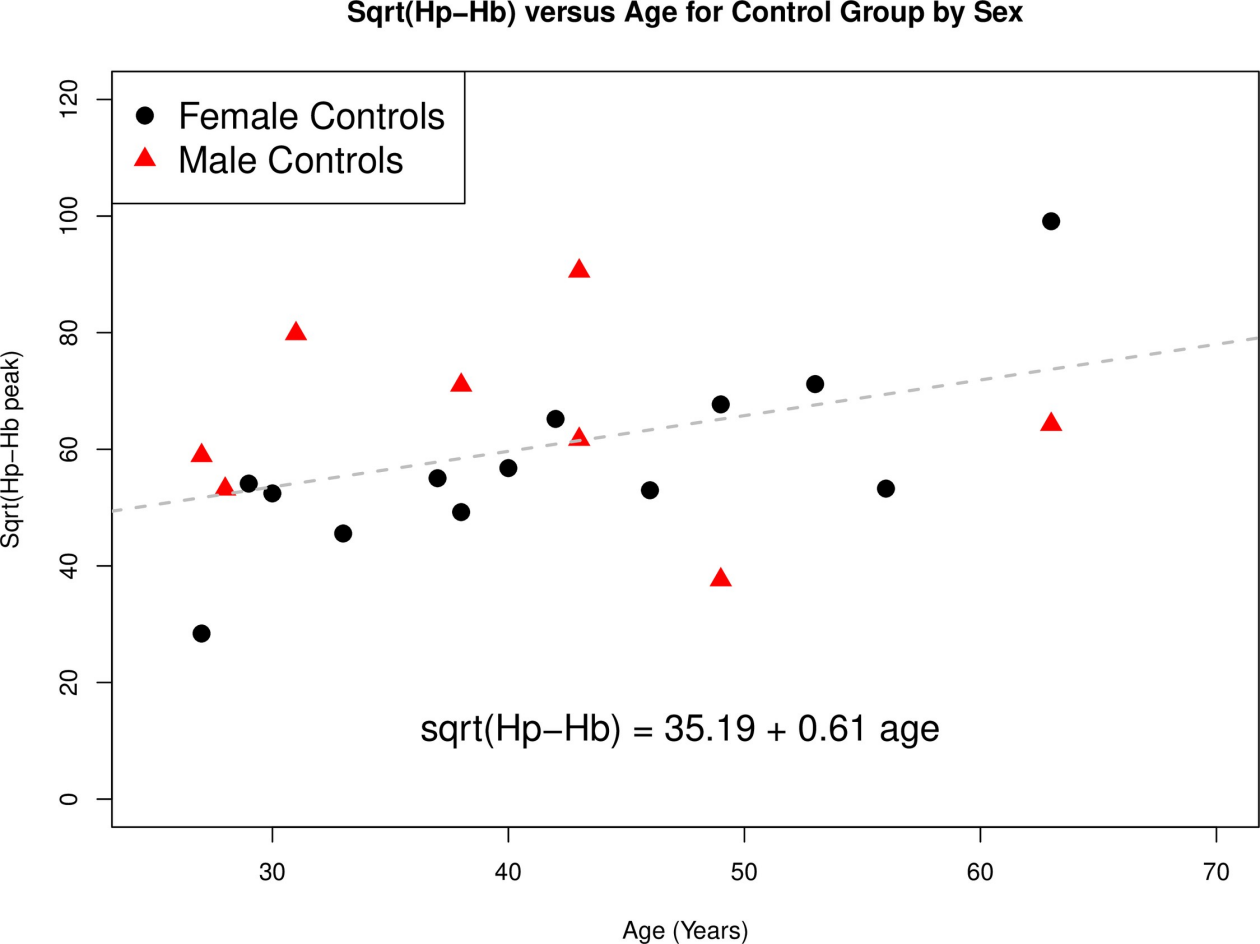
Plot of the age of the control participants (x-axis) against the square root of the obtained Fe-peak areas corresponding to the Hp-Hb complex (y-axis). The regression line is for all controls (adjusted R^2^ of 0.14, F(1,19) = 4.12, p = 0.057) and depicts a positive association between the age of the individual and the Hp-Hb peak area.

When we investigated if sex was a contributing factor to the increased Hp-Hb concentration with age we observed that this trend is mostly driven by females (Fig 3).

### 3.3. Cu and Zn-specific chromatograms of plasma samples obtained by SEC-ICP-AES

**Fig 4:**
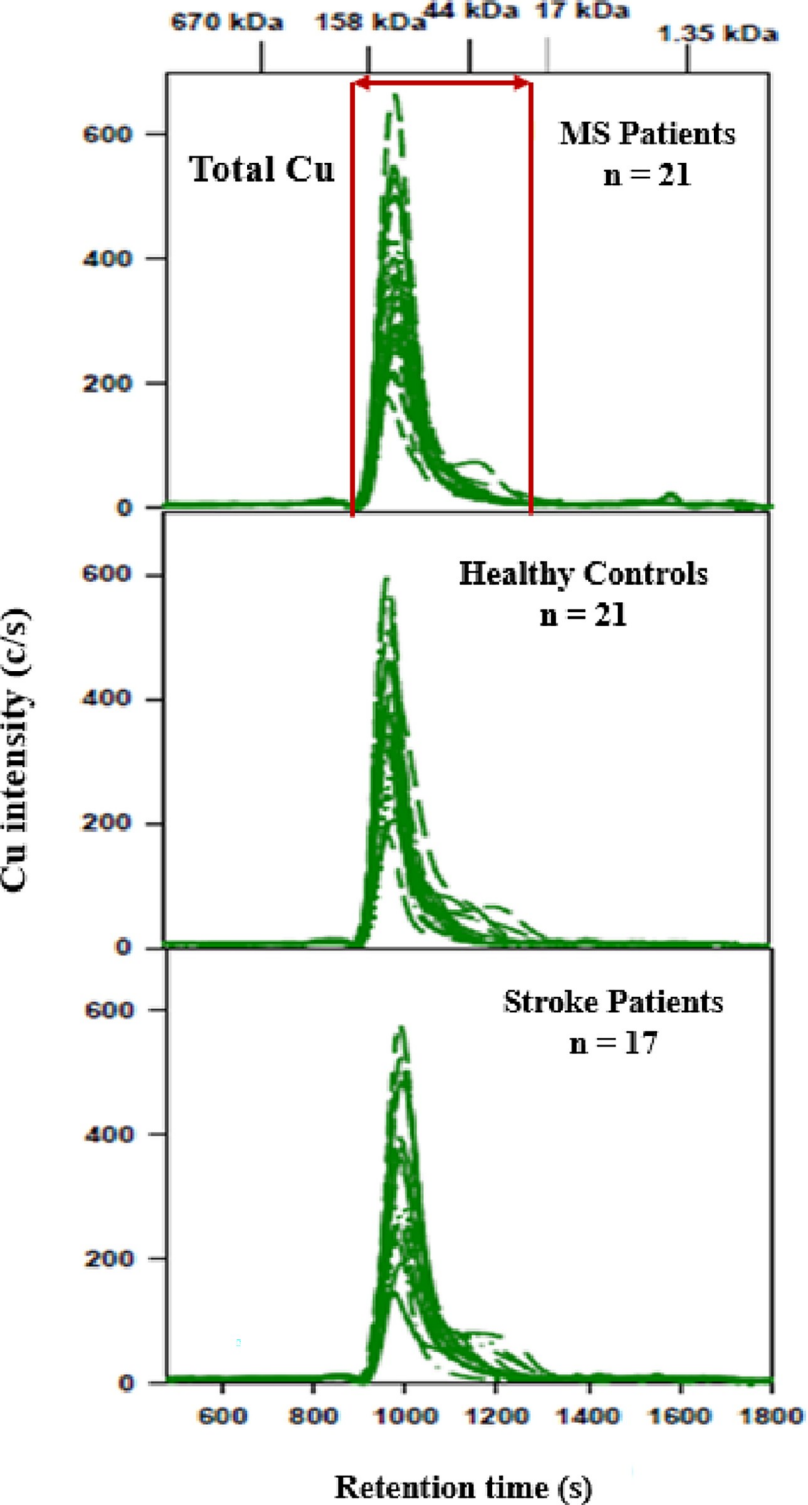
Cu-specific chromatograms obtained after the analysis of plasma from MS participants, AIS participants and healthy controls. Column: Superdex 200 Increase 10/300 GL (30 x 1.0 cm I.D., 8 μm particle size), Temperature: 22°C, Mobile phase: 150 mM PBS buffer (pH 7.4), Flow rate: 0.75 mL/min, Injection volume: 500 μL. ICP-AES detection at 324.75 nm (Cu). Retention times of molecular weight markers are depicted on top. 750 μL of plasma was diluted with 250 μL of PBS buffer and 500 μL of the obtained solution were injected on the SEC column.

**Fig 5:**
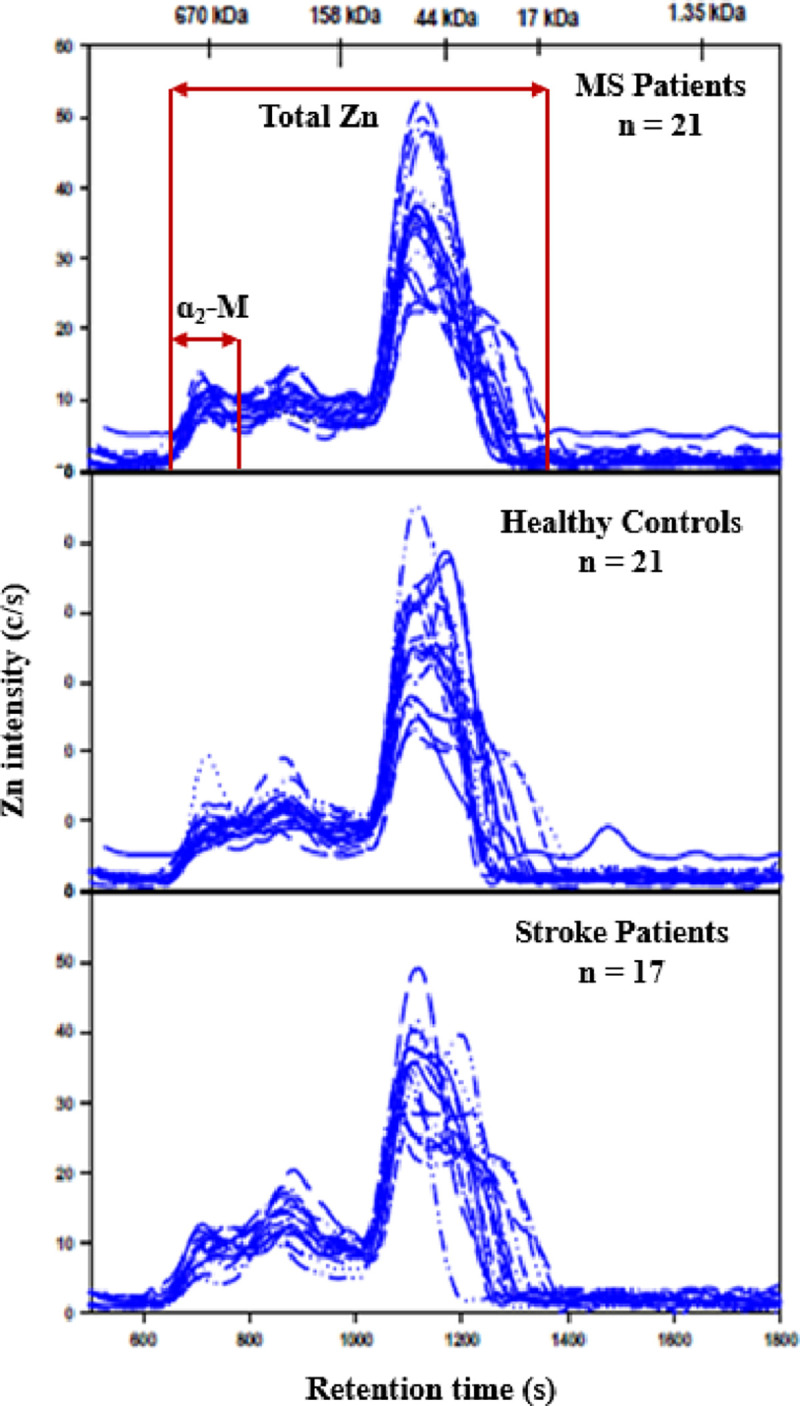
Zn-specific chromatograms obtained after the analysis of plasma from MS participants, AIS participants and healthy controls. Column: Superdex 200 Increase 10/300 GL (30 x 1.0 cm I.D., 8 μm particle size), Temperature: 22°C, Mobile phase: 150 mM PBS buffer (pH 7.4), Flow rate: 0.75 mL/min, Injection volume: 500 μL. ICP-AES detection at 213.86 nm (Zn). Retention times of molecular weight markers are depicted on top. 750 μL of plasma was diluted with 250 μL of PBS buffer and 500 μL of the obtained solution were injected on the SEC column.

For each group the Cu-specific chromatograms (Fig 4) and the corresponding Zn-specific chromatograms (Fig 5) displayed a similar number of peaks that were previously observed for the anlaysis of thawed human plasma [19]. Since no statistically significant differences for total Zn (F (2, 56) = 0.60, p = 0.51) and Cu (F (2, 56) = 1.21, p = 0.31) were observed between the groups via ANOVA, the % recoveries for these metals were not calculated. The peak areas for the Cu peak that corresponds to Cp (Table 3) and the Zn peak that corresponds to α_2_M (Table 4) did not reveal any statistically significant differences between the groups as was evidenced by a one-way analysis of the variance (ANOVA).

**Table 3:** Mean Cu area values, standard deviations and minimum/maximum values obtained for the analysis of serum samples by SEC-ICP-AES (outliers removed).

Peak Area	Groups		
	Control	MS	AIS
**Total Cu**			
n*	21	20	17
Mean (SD^#^)	30,075 (9,809)	31,757 (10,033)	27,622 (11,396)
Min; Max	13,087; 48,306	16,057; 51,853	11,754; 47,279

* number of individuals per group.

^#^ standard deviation.

**Table 4:** Mean Zn area values, standard deviations and minimum/maximum values obtained for the analysis of serum samples by SEC-ICP-AES for total Zn and ɑ_2_M (outliers removed).

Peak Area	Groups		
	Control	MS	AIS
**Total Zn**			
n*	19	21	17
Mean (SD^#^)	9,143 (1,256)	8,498 (1,370)	9,160 (1,595)
Min; Max	6,796; 11,105	5,825;11,163	5,279; 11,432
**ɑ**_**2**_ **-M**			
n	21	20	16
Mean (SD)	981 (370)	972 (257)	890 (275)
Min; Max	340; 1,794	391; 1,461	360; 1,386

* number of individuals per group.

^#^ standard deviation.

## 4. Discussion

Blood plays a critical role in transporting essential metals after their absorption from the GI tract to internal organs, but it also receives metal species shed either from organs and/or red blood cells [5, 34]. The plasma protein haptoglobin (Hb), for example, regulates hemoglobin (Hb) clearance from the circulation by the macrophage-specific receptor CD163 [14] and thus prevents Hb-mediated severe consequences on the kidneys [35]. The analysis of human blood plasma with SEC-ICP-AES has previously allowed the unequivocal identification of ~7 Cu, Fe and Zn metalloproteins [19]. Related analytical methods have been shown to be potentially useful to diagnose certain neurological disorders [8, 22, 26]. To further evaluate the utility of major Fe, Cu and Zn metalloproteins for disease diagnosis, progression and/or severity, we conducted a pilot study to analyze plasma from AIS participants, MS participants and healthy age-matched controls. We note that owing to the small sample size of participants in our study, the statistical power of any observed differences between the groups will inherently be moderate.

Initial analysis showed significantly elevated Hp-Hb in the AIS group, but when the more advanced age of this group was taken into account, no significant difference was found. Age and sex matching was attempted but few young AIS participants were admitted to hospital during the two year recruitment period so only the healthy controls and participants with MS were age and sex matched. While we did not observe statistically significant differences between the metal peak areas corresponding to Cp, α_2_M, Tf and the Hp-Hb complex between the groups (Figs 1–3, Tables 2–4), we observed in three individuals in each group a Hp-Hb complex which contained the Hp 1–1 isoform, whereas the remaining individuals contained the Hp 1–2 and/or the Hp 2–2 isoform. This observation is in accord with our previous studies where we have shown that different Hp isoforms form complexes with Hb that have different size and therefore different retention times [19].

We also investigated if the Hp-Hb peak correlated with age and indeed observed that only age was close to being statistically significant (p = 0.05) in a linear regression model that included the participant groups and the square root of the Fe-peak areas corresponding to the Hp-Hb complex. We note that the observed increased plasma concentration of Hp-Hb complexes with age does not allow us to identify if the lysis of red blood cells occurred *in vivo* or *in vitro* (i.e. during blood collection). Interestingly, the increase of the Hp-Hb concentration in plasma was largely driven by females. There are two possible explanations for our findings. The first is that the release of free Hb from red blood cells increases with age, possibly due to an increase in their fragility [36]. Since it has been reported that the average time for removal of the Hp-Hb complex from blood plasma by macrophage-specific receptor CD163 is 20 min [35], an alternative explanation is that the clearance process of Hp-Hb complexes from the bloodstream itself decreases with age. No previous studies have quantified the Hp-Hb complex in human plasma as a function of age.

Since the primary function of Hp in plasma is to strongly bind Hb to prevent highly toxic free Hb from reaching the kidneys, our results indicate that the release of Hb from aging red blood cells [37, 38] is a likely explanation for the increased Hp-Hb concentration with age. Although we did not quantify free Hp in plasma, it has been previously shown by Zhang et al. that the plasma Hp concentration, but not the Hp isoform, was linked to the outcome after AIS [39]. In addition, significantly elevated levels of free Hp have been reported in serum of aged rats and humans [15] and an increased plasma concentration of free Hp has been linked to neurodegenerative diseases [40, 41], stroke [41] and diabetes [42] as well as the Hp isoform in serum and type 2 diabetes patients [43].

## 5. Conclusion

Blood plasma plays a critical role in transporting essential metals after their absorption from the GI tract to internal organs, but it also receives metals species that shed either from organs and/or red blood cells as a consequence of disease processes. To evaluate the utility of metalloproteins as disease biomarkers we conducted a pilot study and analyzed plasma samples from AIS and MS participants and age matched healthy controls for Fe, Cu and Zn metalloproteins. While we did not observe statistically significant differences between the obtained average Cu, Zn and Fe peak areas corresponding to Cp, α_2_M, Tf and a Hp-Hb complex between the groups, plotting the age of all participants against the square root of the Hp-Hb peak area revealed an increased concentration of the Hp-Hb complex in plasma with age that is predominantly driven by women. These results imply that either the biomolecular mechanisms that mediate the clearance of the Hp-Hb complex from the bloodstream slow down with age or that the fragility of red blood cells increases with age, which is associated with an increased influx of Hb into blood plasma and a higher steady state concentration of the Hp-Hb complex therein. Considering the emerging role that red blood cells may play in certain disease processes [44] and given the conceptual link between the dominant Hp isoform in serum and certain neurodegenerative processes [36, 40, 42, 45], the analysis of plasma/serum from patients with distinct neurodegenerative diseases has the potential to uncover novel metal-based biomarkers. The inherent potential that the analysis of plasma/serum for iron metalloproteins may offer to facilitate disease diagnosis is complemented by the development of analytical methods which not only require much smaller volumes for metalloprotein analysis (50 μL versus 500 μL in this study), but also uses equipment that could be operated by technicians in clinical biochemistry laboratories [7].

## Supporting information

S1 Fig Fe recovery of rabbit plasma after SEC-ICP-AES analysis. (TIF)

S2 Fig Zn recovery for rabbit plasma after SEC-ICP-AES analysis. (TIF)

S3 Fig SEC-ICP-AES derived area counts for the Fe peak corresponding to the Hp-Hb complex in the three groups. (TIF)
