# Supplementary figures and images for: Quantification of human plasma metalloproteins in multiple sclerosis, ischemic stroke and healthy controls reveals an association of haptoglobin-hemoglobin complexes with age
Source: PLoS One. 2022 Jan 12;17(1):e0262160. doi: 10.1371/journal.pone.0262160 (PMC8754309; doi:10.1371/journal.pone.0262160)

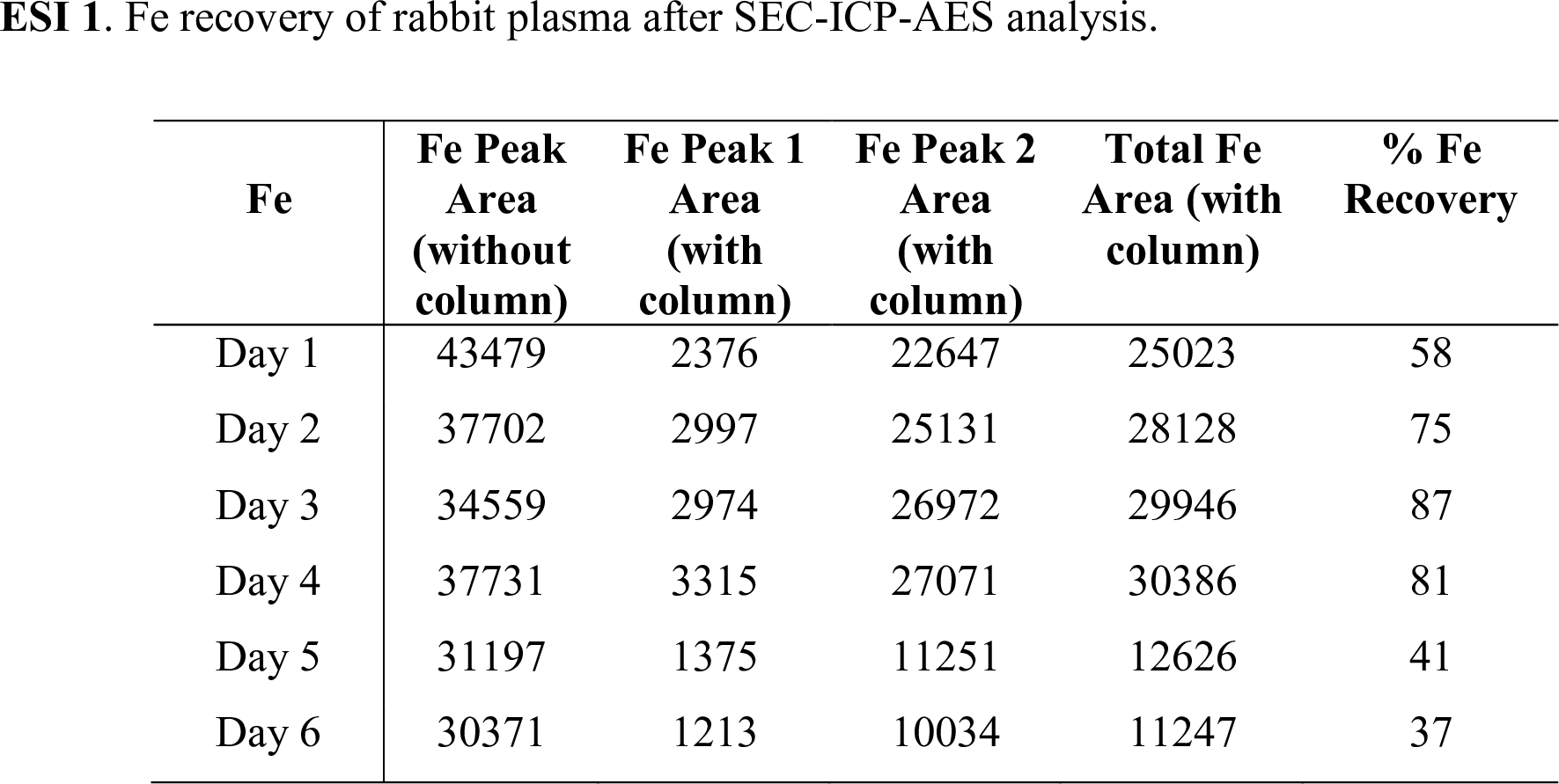

Supplement: S1 Fig — (TIF) [file pone.0262160.s001.tif]

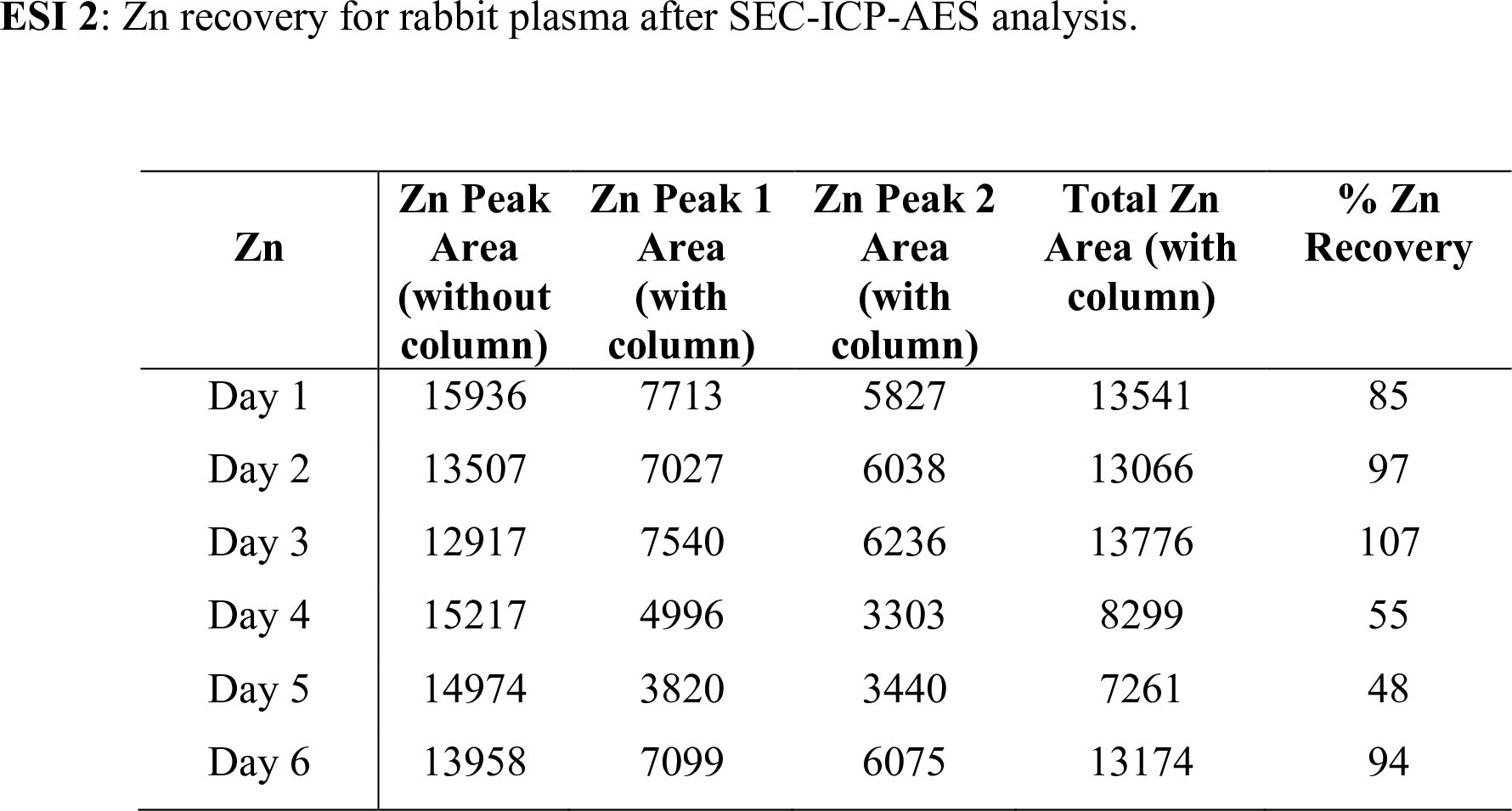

Supplement: S2 Fig — (TIF) [file pone.0262160.s002.tif]

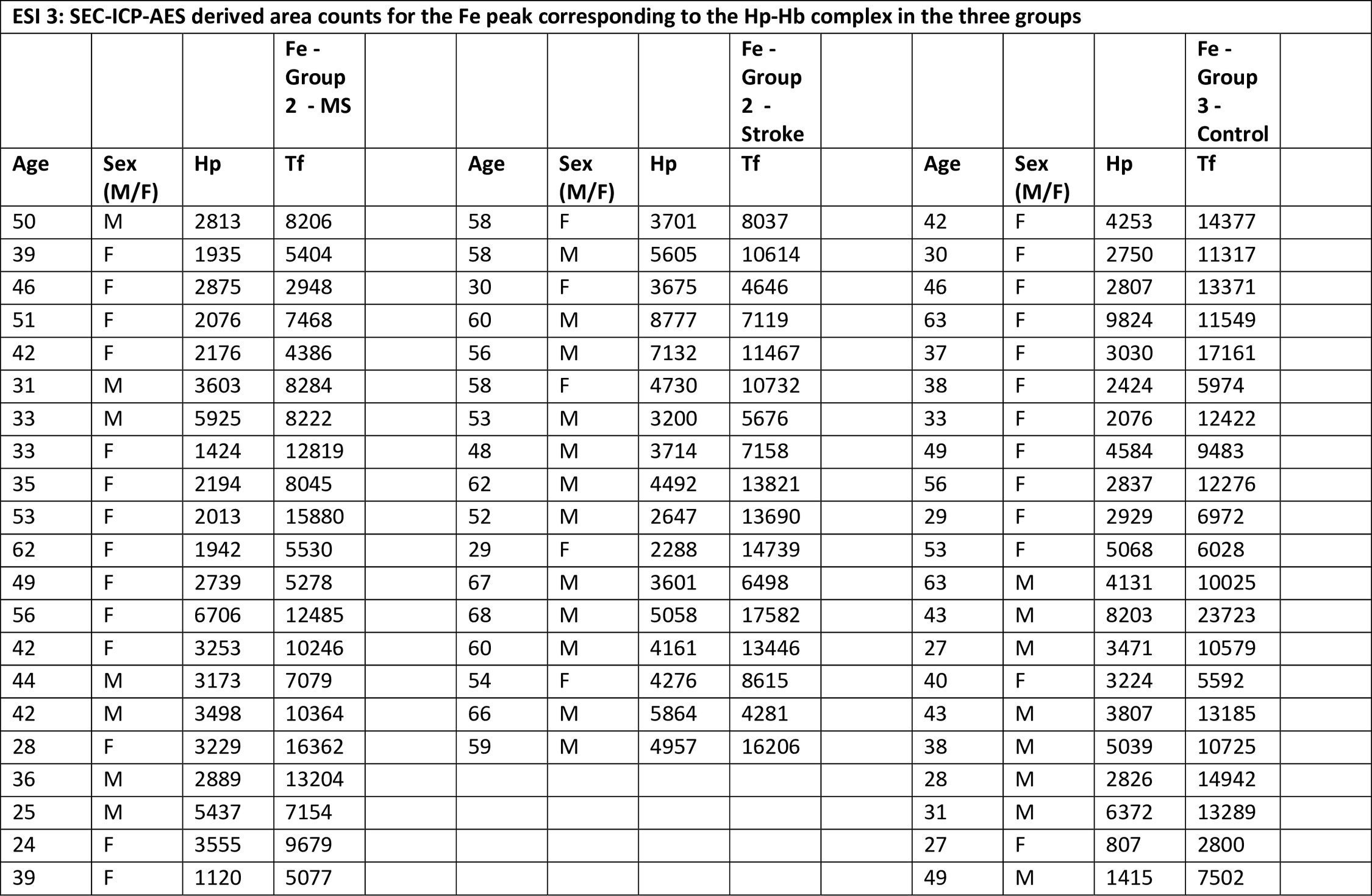

Supplement: S3 Fig — (TIF) [file pone.0262160.s003.tif]
